# DSM-IV post-traumatic stress disorder among World Trade Center responders 11–13 years after the disaster of 11 September 2001 (9/11)

**DOI:** 10.1017/S0033291715002184

**Published:** 2015-11-25

**Authors:** E. J. Bromet, M. J. Hobbs, S. A. P. Clouston, A. Gonzalez, R. Kotov, B. J. Luft

**Affiliations:** 1 Department of Psychiatry, Putnam Hall-South Campus, Stony Brook University, Stony Brook, NY, USA; 2 Program in Public Health and Department of Preventive Medicine, Stony Brook University, Stony Brook, NY, USA; 3 Department of Medicine, Stony Brook University, Stony Brook, NY, USA

**Keywords:** 9/11, Disaster responders, exposure, post-traumatic stress disorder, psychosocial well-being, World Trade Center

## Abstract

**Background:**

Post-traumatic symptomatology is one of the signature effects of the pernicious exposures endured by responders to the World Trade Center (WTC) disaster of 11 September 2001 (9/11), but the long-term extent of diagnosed Diagnostic and Statistical Manual of Mental Disorders, 4th edition (DSM-IV) post-traumatic stress disorder (PTSD) and its impact on quality of life are unknown. This study examines the extent of DSM-IV PTSD 11–13 years after the disaster in WTC responders, its symptom profiles and trajectories, and associations of active, remitted and partial PTSD with exposures, physical health and psychosocial well-being.

**Method:**

Master's-level psychologists administered sections of the Structured Clinical Interview for DSM-IV and the Range of Impaired Functioning Tool to 3231 responders monitored at the Stony Brook University World Trade Center Health Program. The PTSD Checklist (PCL) and current medical symptoms were obtained at each visit.

**Results:**

In all, 9.7% had current, 7.9% remitted, and 5.9% partial WTC-PTSD. Among those with active PTSD, avoidance and hyperarousal symptoms were most commonly, and flashbacks least commonly, reported. Trajectories of symptom severity across monitoring visits showed a modestly increasing slope for active and decelerating slope for remitted PTSD. WTC exposures, especially death and human remains, were strongly associated with PTSD. After adjusting for exposure and critical risk factors, including hazardous drinking and co-morbid depression, PTSD was strongly associated with health and well-being, especially dissatisfaction with life.

**Conclusions:**

This is the first study to demonstrate the extent and correlates of long-term DSM-IV PTSD among responders. Although most proved resilient, there remains a sizable subgroup in need of continued treatment in the second decade after 9/11.

## Introduction

Responders to the World Trade Center (WTC) disaster of 11 September 2001 (9/11), particularly the men and women who were at the site on 11 September, were exposed to emotionally horrifying events and environmental toxins from multiple gases and fine airborne particulate matter from the collapse of the towers. The responders who participated in the rescue, recovery and clean-up operations included experienced workers with extensive training, such as police and firefighters, and non-traditional responders with no disaster training, such as construction workers, electricians, and transportation and utility workers (Dasaro *et al.* 2015). In the aftermath of the attacks, two programs were established to monitor responders' health and treat WTC-related conditions, one for police and non-traditional responders (World Trade Center Health Program; WTCHP) and one for New York City firefighters. In addition, the New York City Department of Health established the WTC Health Registry to track the health and well-being of individuals directly exposed to the collapse of the towers or its immediate aftermath.

All three programs obtained serial data on post-traumatic stress symptoms from the PTSD Checklist (PCL) (Blanchard *et al.* 1996). During the first decade after 9/11, 5–23% of responders had PCL scores suggestive of possible post-traumatic stress disorder (PTSD) (Liu *et al.* 2014), with higher rates among non-traditional compared with professional responders (Perrin *et al.* 2007; Ozbay *et al.* 2013). Trajectory analyses for responders who made three monitoring visits to the WTCHP found that 5.3% of police and 9.5% of non-traditional responders had chronically elevated symptoms, and 8.4% and 12.4%, respectively, experienced a reduction in symptom severity (Pietrzak *et al.* 2014). A recent analysis of responders in the WTC Health Registry found that half of police responders with probable PTSD in the first few years after 9/11 continued to have probable PTSD at 10–11 year follow-up (Cone *et al.* 2015).

WTC exposures, particularly being in the dust cloud and death of colleagues, were significantly associated with self-report PTSD symptoms (e.g. Perrin *et al.* 2007; Yip *et al.* 2015) independent of demographic and other risk factors (Friedman *et al.* 2013). In addition, consistent with the broader literature (O'Toole & Catts, 2008; McFarlane, 2010; Pacella *et al.* 2013), PTSD symptom severity was significantly associated with responders' physical health, particularly respiratory symptoms, a hallmark medical outcome of WTC exposures (Webber *et al.* 2011; Wisnivesky *et al.* 2011; Luft *et al.* 2012; Nair *et al.* 2012; Friedman *et al.* 2013; Pietrzak *et al.* 2014; Cone *et al.* 2015; Kotov *et al.* 2015). The temporal order has not been firmly established, but recent evidence suggests that PTSD symptoms drive the relationship rather than the converse (Kotov *et al.* 2015). Far fewer WTC responder studies have addressed the relationships of PTSD symptoms with psychosocial well-being (e.g. Stellman *et al.* 2008; Schwarzer *et al.* 2014) even though PTSD symptoms are an impediment to quality of life (Koenen *et al.* 2008) and increase the risk of depression (Breslau *et al.* 2000; Stander *et al.* 2014).

All of the studies described above are based on a probable diagnosis of PTSD from the PCL. In contrast, most other research on trauma established the diagnosis of PTSD from structured or semi-structured diagnostic interviews. Indeed, a longitudinal study of returning National Guard soldiers found that the best PCL cut-scores produced 65–76% false-positive errors in relation to a Diagnostic and Statistical Manual of Mental Disorders, 4th edition (DSM-IV) PTSD diagnosis (Arbisi *et al.* 2012), and a study of WTC firefighters suggested that the cut-points used by WTC investigators may be too high (Chiu *et al.* 2011). We found three studies of WTC responders that included a diagnostic interview. Two used the Clinician Administered PTSD Scale (CAPS; Blake *et al.* 1995). The first, conducted with mental health relief workers 6–8 months after 9/11, found that 6.4% met criteria for WTC-related PTSD (Zimering *et al.* 2006). The second study focused on utility workers and found that PTSD declined from 14.9% in 2002 to 5.8% in 2008 (Cukor *et al.* 2011). Cukor *et al.* (2011) also reported that partial PTSD, defined as meeting cluster B and either cluster C or D criteria, decreased from 17.9% to 7.7%. The third study administered the Diagnostic Interview Schedule to a large sample of retired firefighters in 2006–2007; 6.5% had current DSM-IV PTSD (Chiu *et al.* 2011). Thus, no study assessed WTC-related DSM-IV PTSD in police, who were critical first responders on 9/11, or in heterogeneous samples of non-traditional rescue/recovery workers, who had no training in disaster response. We also lack adequate information on the clinical profiles of responders with DSM-IV PTSD and the relative effects of persistent and remitted PTSD on health and psychosocial well-being.

To fill these gaps, we assessed a large cohort of WTCHP police and non-traditional responders in 2012–2014 with the Structured Clinical Interview for DSM-IV (SCID; First *et al.* 1996) and the Range of Impaired Functioning Tool (RIFT) (Leon *et al.* 1999) in order to examine: (1) the percentage of responders developing WTC-PTSD; (2) the criterion symptoms most frequently endorsed by active cases; (3) the comparative course of PCL symptoms for responders with active, remitted, partial and no WTC-PTSD; (4) the effects of physical and psychological exposures on long-term WTC-PTSD; and (5) associations of active, remitted and partial WTC-PTSD with current health and psychosocial well-being.

## Method

### Setting

The sample was derived from the Long Island/Stony Brook University WTCHP, the second largest of five Clinical Centers of Excellence in the New York metropolitan area (Dasaro *et al.* 2015). Enrollees with documented WTC experience were enlisted from extensive outreach efforts involving partnerships with volunteer organizations, labor unions and public outlets.

The protocol and consent form for the current study were approved annually by the Committees on Research Involving Human Subjects at Stony Brook University. Written informed consent was obtained. Assessments took place during regularly scheduled monitoring visits between January 2012 and May 2014.

### Participants

Among the 4587 responders monitored during the study period, 23 could not be approached due to inadequate English language skills. Of the remainder, 430 declined, 630 did not have time to complete the interview, and 3504 participated (76.8% of eligible responders). Their demographic and occupational characteristics (Table 1) are in line with those of the clinic population as a whole.

**Table 1.** Characteristics of responders and associations with DSM-IV WTC-PTSD

	No diagnosis (*n* = 2660), *n* (%)	WTC-PTSD (*n* = 571), *n* (%)	OR (95% CI)
Type of responder			
Non-traditional	719 (27.0)	238 (41.7)	1.9 (1.6–2.3)*
Police	1941 (73.0)	333 (58.3)	1.0
Age at 11 September 2001			
⩾40 years	1051 (39.5)	230 (40.3)	1.0 (0.9–1.2)
<40 years	1609 (60.5)	341 (59.7)	1.0
Gender			
Female	206 (7.7)	74 (13.0)	1.8 (1.3–2.4)*
Male	2454 (92.3)	497 (87.0)	1.0
Education^a^			
Less than college	1858 (69.8)	419 (73.4)	1.2 (1.0–1.5)
Bachelor's degree or higher	802 (30.2)	152 (26.6)	1.0
Employment (initial visit)^a^			
Not working	96 (3.7)	83 (14.8)	5.0 (3.7–6.9)*
Retired	281 (10.8)	95 (17.0)	2.0 (1.4–2.6)*
Employed full/part time	2221 (85.5)	381 (68.2)	1.0
Marital status (initial visit)^a^			
Not married	497 (19.0)	140 (25.0)	1.4 (1.1–1.8)*
Married/with partner	2114 (81.0)	421 (75.0)	1.0
Race			
Other	572 (21.5)	113 (19.8)	0.9 (0.7–1.1)
Caucasian	2088 (78.5)	458 (80.2)	1.0
Smoking status^a^			
Current smoker	159 (6.0)	77 (13.7)	2.5 (1.9–3.3)*
Not current smoker	2475 94.0)	484 (86.3)	1.0
Hazardous drinker^a^			
Yes, current	95 (4.3)	72 (14.6)	3.8 (2.8–5.3)*
No	2132 (95.7)	420 (85.4)	1.0
Current major depression^a^			
Yes	47 (1.8)	218 (38.2)	34.4 (24.6–48.1)*
No	2612 (98.2)	352 (61.8)	1.0
Number of monitoring visits			
3+	1937 (72.8)	422 (73.9)	1.1 (0.86–1.3)
1–2	723 (27.2)	149 (26.1)	1.0
Mental health treatment since 11 September 2001			
Yes	628 (23.6)	435 (76.2)	10.4 (8.4–12.8)*
No	2032 (76.4)	136 (23.8)	1.0

DSM-IV, Diagnostic and Statistical Manual of Mental Disorders, 4th edition; WTC, World Trade Center; PTSD, post-traumatic stress disorder; OR, odds ratio; CI, confidence interval.

a Missing values.

* *p* ⩽ 0.001.

### PTSD

#### DSM-IV PTSD diagnosis

Master's level psychologists were trained to administer the SCID PTSD module without skip-outs (First *et al.* 1996) with interval instructions (worst episode of symptoms since 11 September 2001). SCID items were modified to assess PTSD symptoms in relation to traumatic WTC exposures (criterion A). Before conducting the assessment, the interviewers reviewed participants' occupational and medical histories in order to facilitate rapport and enhance accurate interpretation of responses, particularly to physiological items. Inter-rater agreement for 55 independently rated audiotapes was very good (*κ* = 0.82).

PTSD diagnoses were considered active if current in the past month, remitted if criteria were met in the past only, and partial if DSM-IV criteria were not met but at least one symptom in the B, C and D clusters was endorsed (Breslau *et al.* 2004).

#### PTSD symptom severity

As noted, at each monitoring visit, responders completed the PCL-S (specific version) (Blanchard *et al.* 1996), a 17-item self-report measure modified to assess symptoms over the past month ‘in relation to 9/11’. Severity is rated on a scale from 1 (not at all) to 5 (extremely). The PCL has good convergent validity and internal consistency (Wilkins *et al.* 2011). In the present sample, the internal consistency was excellent (*α* = 0.96). Of note, only 58.8% of responders with active WTC-PTSD had a PCL score indicating possible PTSD (⩾50; Terhakopian *et al.* 2008).

### WTC exposures

Exposure was systematically assessed at the first monitoring visit (Dasaro *et al.* 2015). Six variables were included in the analysis: caught in the dust cloud; early arrival (e.g. on 11 or 12 September; of those arriving later, only 84 arrived after 30 September); duration of work (⩾19 days in September, which was the top quartile, *v.* fewer days); exposure to human remains; knowing someone who was injured; and death of colleagues, friends or family in the tower collapse. A three-level severity index was created to define no/low (0–2), intermediate (3–4) and high (5–6) exposure levels. In the high exposure category, 98.6% lost a colleague or loved one.

### Health and psychosocial well-being

Health variables were: (1) lower respiratory symptoms (cough, wheezing, chest tightness and/or shortness of breath) assessed by medical staff and categorized as WTC-related if absent before or significantly worse following 9/11 and not due exclusively to a cold or similar infection (Luft *et al.* 2012); (2) body mass index ⩾35 kg/m^2^ (e.g. class II obesity or higher) calculated from height and weight measurements; and (3) fair/poor/very poor self-rated health (*v.* good/very good) (Idler & Benyamini, 1997). Psychosocial well-being was determined from interviewer ratings on the RIFT of life satisfaction, relationships with friends and social network involvement (Leon *et al.* 1999). Items were dichotomized into fair/poor/very poor life satisfaction *v.* good/very good, mild/moderate/severely impaired relationships with friends *v.* satisfactory/non-impaired, and fair/poor social network involvement *v.* good/very good.

### General risk factors

These included type of responder [police *v.* non-traditional (primarily construction workers)], age on 9/11 (<40 *v.* ⩾40 years), gender, education (⩾bachelor's degree *v.* lower), occupational status at initial monitoring visit (employed full or part time, retired, not working), marital status at initial visit (married/with partner *v.* separated/divorced/widowed), race (Caucasian; other), current cigarette smoking (Welch *et al.* 2015), current hazardous drinking (⩾8 on the Alcohol Use Disorders Identification Test; AUDIT-10) (Bohn *et al.* 1995) and current DSM-IV major depressive episode (assessed with the SCID). Two service use variables were also included: number of prior monitoring visits and mental health treatment since 9/11 (self-reported use of mental health services at either the initial monitoring visit or study assessment or filing a psychotherapy treatment claim with the WTCHP).

### Statistical analysis

The analyses focused on 3231 Stony Brook responders (92.2% of the sample) with complete information on PTSD, dust cloud exposure and loss of colleagues in the tower collapse. We calculated the percentage of Stony Brook responders who developed PTSD and also estimated the rate for the WTCHP general cohort (*n* = 33 076) by applying weights (Winship & Radbill, 1994) to adjust for the distribution of males (85.6%), Caucasians (56.7%) and police (49%) in the cross-center cohort (Dasaro *et al.* 2015).

Relationships of exposure, health and psychosocial functioning with PTSD were examined using binomial and multinomial logistic regression methods. Analyses were computed with SPSS version 23 (USA).

To examine the trajectories of PTSD symptoms among responders with active, remitted, partial and no DSM-IV PTSD, we used PCL data collected over one to nine monitoring visits, with observations occurring on average 1.6 (s.d. = 1.0) years apart. The analyses incorporated 23 085.8 person-years of observation. We used exact clinic visit dates to calculate years between WTC exposure and observation and years^2^ to examine the potential for non-linear changes over time. Because longitudinal analysis can be biased by repeat measurement and by individual differences in susceptibility or likelihood of reporting PTSD symptoms, we used longitudinal multilevel modeling. We modeled random intercepts, slopes and slopes^2^ to provide the best model fit. We assumed an unstructured covariance matrix to account for regression to the mean and ensure that results were robust to attrition under the assumption that such attrition is not due to an unexpectedly high or low PCL score. We modeled the following equation: 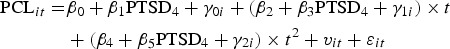 where PCL was measured among individuals (*i*) over time (*t*), PTSD_4_ was the four-category ordinal variable, *v*_*it*_ indicated year of first monitoring visit and was incorporated to model change in sample make-up, and *γ*_*ki*_ refers to random, within-person intercepts and slopes.

### Ethical standards

All procedures contributing to this work comply with the ethical standards of the relevant national and institutional committees on human experimentation and with the Helsinki Declaration of 1975, as revised in 2008.

## Results

The majority of the sample was male (91.3%), Caucasian (78.8%) and worked in law enforcement (70.4%) (Table 1). The median age on 9/11 was 38 years (90% range = 28–50 years). At their first monitoring visit, most were married (79.9%) and employed (82.4%). Less than 10% had current depression, were active smokers or engaged in hazardous drinking. Most had made at least three monitoring visits (73.0%) and one-third (32.9%) received some type of mental health treatment since 9/11.

Nearly one-fifth of the Stony Brook cohort (*n* = 571; 17.7%) developed WTC-PTSD. The estimated weighted rate for the WTCHP general responder cohort was 18.2%. As shown in Table 1, non-traditional responders were twice as likely to develop WTC-PTSD compared with police (24.9% *v.* 14.6%). Among police, the percentage with PTSD was significantly lower (*p* < 0.001) for active duty (12.0%) than retired officers (27.2%); among non-traditional responders, the percentages were similar (21.5% and 20.0%, respectively). Consistent with epidemiological findings, female gender, marital status and employment as well as smoking, hazardous drinking, current depression and mental health treatment were significantly associated with PTSD. As expected, current depression was strongly co-morbid with PTSD in both police and non-traditional responders.

With respect to recency, 9.7% (*n* = 315) of the sample had active PTSD and 7.9% (*n* = 256) were in remission. Thus, slightly more than half of the responders who developed PTSD (55.2%) had active disorder at the time of interview. This was true in both police (53.2%) and non-traditional responders (58.0%). Furthermore, 5.9% (*n* = 191) of the sample had partial PTSD, primarily in the past (two-thirds of partial cases). Partial PTSD was less frequent in police (6.3%) than in non-traditional responders (9.5%) (*χ*^2^ = 7.67, degrees of freedom = 1, *p* < 0.01).

### PTSD symptoms

**Fig. 1.**
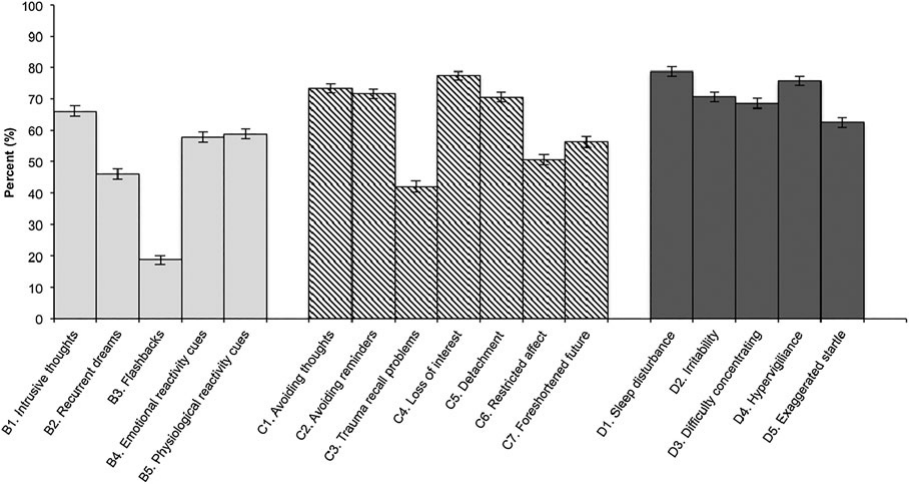
Distribution of Diagnostic and Statistical Manual of Mental Disorders, 4th edition (DSM-IV) post-traumatic stress disorder (PTSD) criteria B (intrusive recollection), C (avoidance/numbing) and D (hyperarousal) symptoms among responders with active World Trade Center PTSD in 2012–2014. Values are percentages, with 95% confidence intervals represented by vertical bars.

Fig. 1 shows the distribution of SCID PTSD symptoms for responders with active PTSD. More than two-thirds reported intrusive thoughts (B1), avoiding thoughts and reminders (C1–2), loss of interest and detachment (C4–5), and four of the five hyperarousal symptoms (D1–4). The least frequently reported symptoms were recurrent dreams and flashbacks (B2–3) and inability to recall aspects of the trauma (C3).

**Fig. 2.**
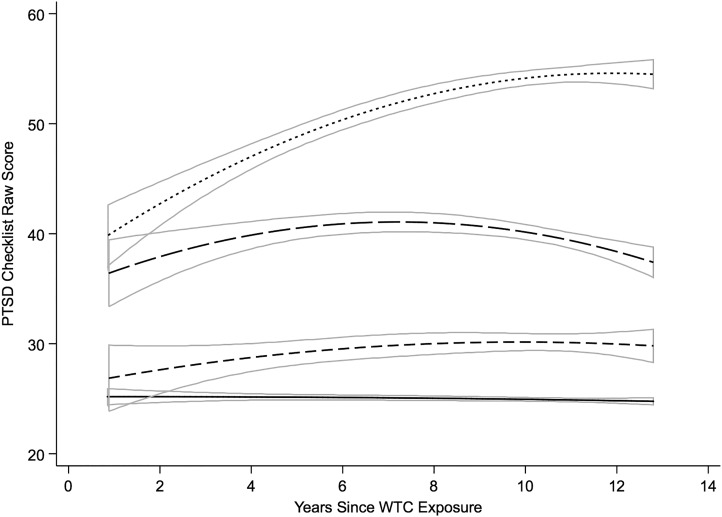
Predicted trajectories derived from longitudinal models of PTSD Checklist data for responders with no history of World Trade Center (WTC) post-traumatic stress disorder (PTSD) (––), and partial (- - -), remitted (– – –) and active WTC-PTSD (· · ·). The boxes outlined in solid gray represent 95% confidence intervals.

The trajectories of PCL symptoms across monitoring visits for responders with active, remitted and partial PTSD are shown in Fig. 2. The overall longitudinal associations are provided in online Supplementary Table S1, and fit statistics and reasoning for our model choice are provided in online Supplementary Table S2. We note that time of the first monitoring visit was not significantly associated with initial PCL score (online Supplementary Table S1). The random-effects intercepts suggest that there were individual differences in PTSD symptom severity, while the significant slope and slope^2^ covariates suggest that growth in PCL scores was heterogeneous over time. There was also a negative covariance between intercepts and slope and slope^2^, suggesting that those with higher scores were more likely to experience symptom reduction over time than those with lower capability. The strong negative correlation between individual slopes and slope^2^ suggests that those who experienced more rapid increases in PCL scores also tended to experience more deceleration, possibly indicative of floor and ceiling effects in the sensitivity of the PCL.

Responders with active WTC-PTSD had the highest PCL scores at the initial monitoring visit which increased over time and then plateaued (Fig. 2). Responders with remitted PTSD had lower initial PCL scores that also increased over time but then decelerated. In contrast, responders with partial PTSD were similar initially to those with no history of PTSD, but their PCL scores increased in a linear fashion over time while those without PTSD retained their low PCL scores.

### Exposure and PTSD

One-quarter of the sample arrived on 11–12 September (25.2%), and one-quarter were in the dust cloud (23.0%). More than half were exposed to human remains (71.5%), knew someone who was injured on 9/11 (56.0%), or suffered a loss (68.1%), primarily of colleagues (78.1% of deaths). One-quarter (23.7%) experienced five to six exposures. Police responders were significantly more likely (*p* < 0.001) to report each exposure and were twice as likely as non-traditional responders to be in the high exposure category (28.4% *v.* 12.4%).

Because responder type was significantly associated with both exposure and PTSD, we analysed the associations of exposure with PTSD separately for police and non-traditional responders. Table 2 shows that among police, each exposure increased the risk of WTC-PTSD and of active, remitted and, to a lesser extent, partial PTSD. The largest odds ratios were for exposure to human remains and experiencing five to six exposures. Among non-traditional responders, the associations of exposures with WTC-PTSD and with active and remitted PTSD were also significant, albeit somewhat weaker. Associations with partial PTSD were by and large non-significant with the exceptions of arrival on 11–12 September, which increased the risk of partial PTSD three-fold, and being in the highest exposure category, which more than doubled the risk.

**Table 2.** Relationships of exposures to WTC-PTSD compared with partial/no PTSD, and with active, remitted and partial PTSD compared with no PTSD

	WTC-PTSD	Active PTSD	Remitted PTSD	Partial PTSD
	OR (95% CI)	AOR (95% CI)	AOR (95% CI)	AOR (95% CI)
Police responders (*n* = 2274)				
Exposure type				
Arrival 11–12	1.9 (1.3–2.6)***	1.9 (1.2–3.0)**	2.0 (1.2–3.2)**	1.9 (1.1–3.3)*
September				
In dust cloud	1.9 (1.5–2.4)***	1.8 (1.3–2.5)***	2.2 (1.6–3.1)***	1.8 (1.2–2.6)*
Worked >19 days	1.5 (1.2–1.9)***	1.6 (1.1–2.2)**	1.6 (1.1–2.2)**	1.5 (1.0–2.2)*
Human remains	3.0 (2.0–4.6)***	3.6 (2.0–6.5)***	2.6 (1.5–4.6)***	1.4 (0.9–2.3)
Injury	2.1 (1.6–2.7)***	2.1 (1.5–3.0)***	2.2 (1.5–3.1)***	1.5 (1.0–2.2)*
Death	2.4 (1.7–3.4)***	2.5 (1.6–3.9)***	2.4 (1.5–3.8)***	1.3 (0.8–2.0)
Exposure severity (number)				
High (5–6)	3.8 (2.4–6.1)***	4.8 (2.4–9.8)***	3.4 (1.8–6.2)***	2.2 (1.1–4.2)*
Intermediate (3–4)	2.0 (1.2–3.1)***	2.8 (1.4–5.6)**	1.4 (0.8–2.6)	1.5 (0.8–2.8)
No/low (0–2)	1.0	1.0	1.0	1.0
Non-traditional responders (*n* = 957)				
Exposure type				
Arrival 11–12	1.9 (1.4–2.7)***	1.9 (1.2–2.8)**	2.5 (1.5–4.3)***	3.3 (1.7–6.4)***
September				
In dust cloud	2.0 (1.4–2.9)***	1.8 (1.1–2.8)*	2.7 (1.7–4.4)***	1.6 (0.9–3.1)
Worked >19 days	1.3 (0.9–2.0)	1.5 (0.9–2.5)	1.3 (0.7–2.3)	1.8 (0.9–3.3)
Human remains	2.0 (1.4–2.8)***	1.9 (1.2–2.8)**	2.3 (1.4–3.8)***	1.3 (0.8–2.2)
Injury	1.9 (1.3–2.5)***	2.0 (1.4–2.9)***	1.9 (1.2–2.9)**	1.4 (0.8–2.3)
Death	1.6 (1.2–2.1)**	1.9 (1.3–2.8)***	1.3 (0.9–2.0)	1.5 (0.9–2.5)
Exposure severity (number)				
High (5–6)	2.9 (1.8–4.5)***	3.4 (1.9–6.2)***	3.0 (1.6–5.5)***	2.4 (1.2–5.1)*
Intermediate (3–4)	1.5 (1.1–2.1)*	1.9 (1.2–2.9)**	1.2 (0.7–1.9)	1.1 (0.6–2.0)
No/low (0–2)	1.0	1.0	1.0	1.0

WTC, World Trade Center; PTSD, post-traumatic stress disorder; OR, odds ratio; CI, confidence interval; AOR, adjusted odds ratio.

* *p* < 0.05, ** *p* ⩽ 0.01, *** *p* ⩽ 0.001.

### Relationships of WTC-PTSD with health and psychosocial well-being

Table 3 shows the relationships between WTC-PTSD and health and psychosocial well-being. Findings from the unadjusted analyses indicated that WTC-PTSD, especially active PTSD, was significantly associated with each of the health and psychosocial variables. In addition, we found a set of graded relationships across active, remitted and partial PTSD for each variable except severe obesity. After adjusting for exposure severity, demographic characteristics and other PTSD risk factors (smoking, hazardous drinking, depression, mental health treatment), WTC-PTSD was significantly associated with respiratory symptoms, subjective health, life satisfaction and social support, but not severe obesity or poorer relationships with friends. The graded pattern of relationships with active, remitted and partial PTSD was diminished with the exception of life satisfaction, on which responders with active PTSD were more than seven times as likely and those with remitted PTSD more than twice as likely than unaffected responders to be rated as having fair/poor satisfaction. The adjusted odds ratio for partial PTSD with life satisfaction was not significant.

**Table 3.** Associations of WTC-PTSD with health and well-being: binomial and multinomial logistic regression analyses

	Unadjusted analysis						Adjusted analysis^a^			
	WTC-PTSD *v.* partial/none	Active PTSD	Remitted PTSD	Partial PTSD	None	WTC-PTSD *v.* partial/none	Active PTSD	Remitted PTSD	Partial PTSD	None
Variables	OR (95% CI)	aOR (95% CI)	aOR (95% CI)	aOR (95% CI)	Ref	AOR (95% CI)	AOR (95% CI)	AOR (95% CI)	AOR (95% CI)	Ref
Health status										
Respiratory symptoms	2.7 (2.3–3.3)***	4.6 (3.5–5.9)***	1.6 (1.3–2.1)***	1.4 (1.0–1.8)*	1.0	1.7 (1.3–2.2)***	2.6 (1.8–3.8)***	1.4 (1.0–1.9)*	1.3 (0.9–1.8)	1.0
BMI ⩾35 kg/m^2^	1.3 (1.1–1.7)**	1.3 (1.0–1.7)	1.5 (1.1–2.0)**	1.2 (0.8–1.7)	1.0	1.3 (1.0–1.8)	1.1 (0.7–1.8)	1.5 (1.0–2.1)*	1.1 (0.7–1.7)	1.0
Fair/poor health	4.4 (3.6–5.3)***	7.6 (5.8–9.9)***	2.7 (2.1–3.5)***	1.7 (1.3–2.4)***	1.0	1.9 (1.5–2.5)***	2.6 (1.7–3.7)***	1.8 (1.3–2.4)***	1.7 (1.2–2.4)**	1.0
Psychosocial status										
Fair/poor satisfaction	10.5 (8.5–12.9)***	28.1 (20.3–38.9)***	4.8 (3.7–6.3)***	1.8 (1.2–2.5)**	1.0	3.5 (2.7–4.7)***	7.6 (4.9–11.7)***	2.4 (1.7–3.4)***	1.3 (0.8–2.0)	1.0
Impaired friendships	2.4 (1.9–2.9)***	3.7 (2.9–4.8)***	1.2 (0.8–1.6)	0.7 (0.4–1.1)	1.0	1.2 (0.9–1.6)	1.9 (1.2–2.8)**	0.7 (0.5–1.1)	0.7 (0.4–1.1)	1.0
Fair/poor social support	2.5 (2.1–3.1)***	3.7 (2.9–4.8)***	1.7 (1.3–2.2)***	1.4 (1.1–2.0)*	1.0	1.4 (1.2–2.0)**	2.2 (1.5–3.1)***	1.2 (0.9–1.7)	1.4 (0.9–2.0)	1.0
										

WTC, World Trade Center; PTSD, post-traumatic stress disorder; OR, odds ratio; CI, confidence interval; aOR, odds ratio in multinomial regression; ref, reference; AOR, adjusted odds ratio; BMI, body mass index.

a Adjusted for variables significant in Tables 1 and 2: responder type, exposure severity, gender, employment, marital status, smoking, hazardous drinking, current depression and mental health treatment since 11 September 2001.

* *p* < 0.05, ** *p* ⩽ 0.01, *** *p* ⩽ 0.001.

## Discussion

Nearly one-fifth of the WTC responders monitored at the second largest WTCHP developed DSM-IV WTC-PTSD after 9/11, half of whom had active disorder 11–13 years on. The most frequent symptoms in the active PTSD group were avoidance and hyperarousal symptoms, while intrusive recollection symptoms were less commonly endorsed. The longitudinal trajectories of PTSD symptomatology rated on the PCL showed a modestly increasing slope for responders with active PTSD and a decelerating pattern for the remitted group. More than a decade after 9/11, WTC exposures remained strongly predictive of both active and remitted PTSD, especially among police responders. WTC-PTSD was strongly associated with health and psychosocial well-being. While these relationships were attenuated after adjustment for exposure, demographic characteristics and known risk factors for PTSD, including depression, they remained robust more than a decade after 9/11, particularly among responders with active WTC-PTSD.

This is the first study of a broad sample of professional and non-traditional WTC responders designed to examine the extent of DSM-IV WTC-related PTSD based on a clinician-administered diagnostic assessment (SCID) and associations with psychosocial well-being determined from a reliable semi-structured interview (RIFT). The interviewing team was composed of master's-level clinical psychologists who reviewed medical records prior to each interview to enhance the accuracy of the assessments. A key emphasis of the training was distinguishing between symptoms directly related to 9/11 exposures and ones that were secondary to 9/11 physical illnesses, particularly respiratory and gastrointestinal conditions. An additional strength was the inclusion of a comprehensive set of potential confounders, including hazardous drinking, smoking, current DSM-IV depression and mental health service use, in analysing associations of active, remitted and partial PTSD with health and psychosocial well-being.

The findings, however, require independent confirmation. Similar to other major disasters, such as the Amsterdam El Al crash (e.g. Huizink *et al.* 2006), the Oklahoma City bombing (North *et al.* 2002) and the Chernobyl nuclear power plant accident (Loganovsky *et al.* 2008), there is no complete list of WTC responders. The three largest cohorts are the WTCHP, which verifies responder status, the New York City firefighter cohort, requiring annual examinations starting before 9/11, and the WTC Health Registry, where inclusion is based on self-report. Although the percentages reported here should be regarded cautiously, our findings are consistent with those of previous studies of WTC responders and research on other rescue/recovery workers. For example, the proportion of police responders with active (current) PTSD (7.8%) is in line with the estimate for WTC retired firefighters 7 years after 9/11 (6.5%; Chiu *et al.* 2011) and with the global pooled estimate of 10% reported in a recent meta-analysis of a worldwide sample of >20 000 rescue workers (Berger *et al.* 2012). The percentage with post-9/11 PTSD in our sample (17.7%) and the weighted percentage for the entire WTCHP (18.2%) are consistent with the lifetime rate of 18.7% in the National Vietnam Veterans Readjustment Study (NVVRS) theater cohort assessed with the SCID 20–25 years later (Dohrenwend *et al.* 2008). Furthermore, the associations reported here for exposure, demographic and other risk factors are consistent with findings from previous trauma studies (McFarlane, 2010; Del Gaizo *et al.* 2011; Kilpatrick *et al.* 2013). Second, like other large-scale studies (e.g. NVVRS, Schlenger *et al.* 2007; Project VALOR, Rosen *et al.* 2012), we administered the SCID rather than the ‘gold standard’ CAPS primarily because of time constraints. Third, the study was cross-sectional and observational though the inclusion of serial symptom data enriched the clinical results. Fourth, like other WTC studies, we did not have an unexposed comparison group. Last, this report focused exclusively on WTC-PTSD. However, responders were also at increased risk of depression and other anxiety disorders (Fullerton *et al.* 2004; Alexander & Klein, 2009) and were exposed to prior and subsequent stressors. A recent study found an interaction effect of WTC exposure and post-9/11 stressful life events on PTSD symptomatology (Zvolensky *et al.* 2015). Future studies of DSM-IV PTSD in WTC responders will directly address these co-morbidities and sources of stress.

Within the context of these limitations, the current study sheds light on three issues. First, more than a decade after 9/11, the catastrophic exposures continued to have an adverse impact on clinically defined PTSD in both professional and non-traditional WTC responders. Although every disaster is unique, the findings add to the few long-term follow-ups of traumatic events, including Chernobyl clean-up workers (Loganovsky *et al.* 2008), Vietnam veterans (Schlenger *et al.* 2007) and Israeli combat veterans (Solomon *et al.* 2009). The percentage of police with active PTSD (7.8%) was lower than that of non-traditional responders (14.4%) and also lower than the rate of probable PTSD in police in the WTC Health Registry assessed with the PCL (11.0% in 2011–2012; Cone *et al.* 2015). It has been argued that active-duty police minimize their symptoms because of employment-related concerns (Luft *et al.* 2012), and, indeed, a lower percentage of active-duty police had WTC-PTSD compared with retired police officers. Even with this potential bias, the persistence of PTSD is striking, with approximately 50% of affected responders having active PTSD. Interestingly, the percentage with remitted PTSD 12–14 years post-9/11 was comparable with the median time to remission (14 years) for participants with PTSD in the 2007 Australian National Survey of Mental Health and Wellbeing (Chapman *et al.* 2012).

Second, although the diagnosis of PTSD connotes ‘flashbacks’ and ‘nightmares’ in popular culture, these symptoms were far less frequently reported than avoidance and hyperarousal symptoms. Furthermore, the trajectory of PTSD symptoms among responders with active PTSD showed progressively increasing symptom severity, indicating both unmet need for treatment and potential undertreatment for those receiving mental health services. Although ‘gold standard’ treatments, such as cognitive–behavioral therapy and prolonged exposure therapy, were designed to address PTSD symptoms, a recent meta-analysis (Watts *et al.* 2013) showed that these treatments potentially benefit only one in two patients. Even so, given that 50% of responders with PTSD had active disorder, there is a clear need to continue to monitor the cohort and to encourage the group with active PTSD to seek evidence-based treatment.

Third, PTSD was significantly associated with several aspects of health and psychosocial well-being, especially self-reported fair/poor health and reduced life satisfaction. Even after adjustment for exposure severity and other risk factors, the reach of PTSD into other domains of life was clearly shown and was strongest for the group with active PTSD. The current associations with the health variables, especially respiratory symptoms and negative subjective evaluations obtained as part of the monitoring visit, are consistent with the recent national initiative to provide integrated physical and mental health care (Huffman *et al.* 2014). The findings also suggest that a biopsychosocial formulation should be an integral part of treatment planning. In fact, the Stony Brook clinic is modeled on an integrated physical and mental health care system, offering on-site mental and physical health care (Luft *et al.* 2012).

In conclusion, the long-term impact of 9/11 exposures, like combat experiences in military cohorts, is reflected in the substantial percentage with PTSD more than a decade after 9/11 and its strong associations with physical health and psychosocial well-being, especially reduced satisfaction with life. Responders experienced multiple exposures simultaneously and, like other rescue/recovery workers, were at increased risk by virtue of the combination of proximity to the disaster site, duration of work and intensity of the exposures (Benedek *et al.* 2007). The concurrent associations with poorer health and psychosocial well-being were striking, particularly for responders with active PTSD though the remitted group also had a 2-fold increased level of dissatisfaction with life and negative subjective health. These results suggest the need for monitoring programs to consider all aspects of health as defined by the World Health Organization, namely, mental, physical and social well-being. Future longitudinal studies are needed to determine the continued persistence of PTSD in this cohort, the potential for relapse among remitted cases and for delayed onset in the unaffected group, and the extent to which the course of PTSD predicts decrements in health and psychosocial well-being during the second decade after 9/11.

## Supplementary material

For supplementary material accompanying this paper visit http://dx.doi.org/10.1017/S0033291715002184. click here to view supplementary material
