# Supplementary material for: DSM-IV post-traumatic stress disorder among World Trade Center responders 11–13 years after the disaster of 11 September 2001 (9/11)
Source: Psychol Med. 2015 Nov 25;46(4):771–83. doi: 10.1017/S0033291715002184 (PMC4754831; doi:10.1017/S0033291715002184)
Supplement: Supplementary file 1 [file S0033291715002184sup001.doc]

***Supplementary Table S1.*** *Beta coefficients, standard errors, and p-values examining the longitudinal associations* between PTSD status and PCL total score

|  |  | B | SE | p |
| --- | --- | --- | --- | --- |
| PTSD Status | No PTSD | Reference | | |
|  | Partial | -0.025 | 1.906 | 0.989 |
|  | Remitted | 11.774 | 1.841 | <0.001 |
|  | Active | 13.296 | 1.536 | <0.001 |
| Years since WTC exposure | | 0.094 | 0.137 | 0.492 |
| PTSD x Years | No PTSD | Reference | | |
|  | Partial | 1.116 | 0.482 | 0.020 |
|  | Remitted | 1.228 | 0.454 | 0.007 |
|  | Active | 2.349 | 0.382 | <0.001 |
| Years^2^ |  | -0.012 | 0.008 | 0.145 |
| PTSD x Years^2^ | No PTSD | Reference | | |
|  | Partial | -0.052 | 0.029 | 0.077 |
|  | Remitted | -0.089 | 0.027 | 0.001 |
|  | Active | -0.076 | 0.023 | 0.001 |
| Visit number |  | 0.101 | 0.051 | 0.048 |
| Intercept |  | 0.101 | 0.020 | <0.001 |
|  |  |  |  |  |
| Random Effects | |  |  |  |
| Intercepts |  | 11.970 | 0.506 | <0.001 |
| Slope |  | 2.922 | 0.147 | <0.001 |
| Slope^2^ |  | 0.180 | 0.010 | <0.001 |
| Corr(Intercept, Slope) | | -0.631 | 0.033 | <0.001 |
| Corr(Intercept, Slope^2^) | | 0.462 | 0.048 | <0.001 |
| Corr(Slope, Slope^2^) | | -0.946 | 0.007 | <0.001 |
|  |  |  |  |  |
| Residuals |  | -0.946 | 0.007 |  |
|  |  |  |  |  |
| Pseudo-R^2^ |  |  | 0.138 | <0.001 |

Note: PCL=Posttraumatic Stress Disorder Checklist

***Supplementary Table S2.*** *Fit statistics for longitudinal modeling supporting model choice*

|  | Model 1 Null Model | Model 2 Random Intercepts | Model 3 Random Linear Slopes | Model 4 Random Quadratic Slopes | Model 5 Correlated Random Effects (Shown) |
| --- | --- | --- | --- | --- | --- |
| AIC | 119327 | 106084 | 105413 | 105388 | 105006 |
| Change AIC |  | -13243 | -671 | -25 | -383 |

**Note**: Akaike’s information criteria provided; smaller AIC indicates a better model. Nested models do not incorporate predictors, only fixed and random intercepts and slopes. Each model incorporates sections shown in the following equation:
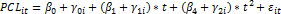
. Model 1 is a null model with no covariates and thus only incorporates only
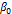
. Model 2 incorporates individually-specific estimates of average PCL scores (
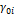
). Model 3 further incorporates linear slope estimates (specifically,
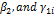
). Model 4 further incorporates
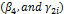
. Finally, model 5 additionally accounts for the associations between individual-level intercepts, slopes, and quadratic slopes (specifically,
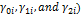
. Because Model 5 shows the smallest AIC, it is the best-fitting model and is relied upon for these analyses.
